# Feeling lonely in the online crowd: what TikTok tells us about young people and loneliness

**DOI:** 10.1093/heapro/daaf094

**Published:** 2025-07-07

**Authors:** Bethan Harries, Marco Zenone, Greg Hartwell

**Affiliations:** Faculty of Public Health & Policy, London School of Hygiene and Tropical Medicine, Keppel Street, London, WC1E 7HT, United Kingdom; School of Public Policy and Global Affairs, University of British Columbia, 1855 West Mall, Vancouver, BC, V6T 1Z2, Canada; Faculty of Public Health & Policy, London School of Hygiene and Tropical Medicine, Keppel Street, London, WC1E 7HT, United Kingdom

**Keywords:** public health, social media, TikTok, loneliness, mental health, digital health

## Abstract

Loneliness is a pressing public health issue, with young people particularly affected. Given the widespread use of TikTok among younger demographics, the platform plays an influential role in shaping perceptions of health topics. This study examines the self-perceived causes and experiences of loneliness expressed by TikTok users. We built a dataset of the most popular, publicly available English-language TikTok videos globally that used the hashtag #lonely and performed a qualitative content analysis to assess (i) video tone, (ii) self-expressed identities related to loneliness, (iii) unmet desires expressed as the cause of loneliness, (iii) absences expressed as the cause of loneliness, and (v) changes in life circumstance as the cause of loneliness. The included videos (*n* = 184) generated over 687 million views. Absence (69.6%) was the prevailing theme of self-perceived causes of loneliness. This absence was mostly attributed to a lack of quality in a person’s relationships (33.7%) or having no relationship (32.1%). Mental health (26.1%) was the most cited expression of identity related to the experience of loneliness. The overall sentiment of the videos was negative (83.2%). No videos were made by healthcare professionals or healthcare organizations offering health information or support. TikTok is an accessible, underutilized, and valuable data resource to understand the public portrayal and sentiment of pressing health topics in young people. Results reveal a prevalence of negatively toned videos, primarily expressing absence as a root cause of loneliness.

Contribution to Health Promotion

- This study contributes to health promotion by examining the self-perceived causes and experiences of loneliness among TikTok users, identifying relational absence as a predominant self-perceived cause.
- The absence of content from healthcare providers reveals a critical gap in public health engagement.
- This research is valuable for informing future public health initiatives, by providing insights into TikTok users’ experience of loneliness and emphasizing the need for targeted resources to engage young audiences and address this pressing public health issue.

## INTRODUCTION

Loneliness is now recognized as a priority area for global public health, and young adults are particularly prone to being lonely (Fardghassemi and Joffe 2022, Berlingieri *et al*. 2023, Jaffe 2023, Priya Giri and Dubey 2023, The Lancet 2023). The action of governments, and other multilateral organizations show how seriously the issue is taken: Vivek Murthy, the former United States Surgeon General, warned that the country faces an epidemic of loneliness (Jaffe 2023), while the United Kingdom appointed the first-ever Minister for Loneliness in 2018, and Japan followed suit in 2021. In 2022, the European Commission launched an initiative to understand the effects of loneliness across its member states (Berlingieri *et al*. 2023). Even so, little is known about how young people view the causes of their own loneliness (Berlingieri *et al*. 2023). Better information would help inform public health interventions to reduce loneliness and its health impacts. Exploring self-perceived causes of loneliness in this demographic is therefore a research priority.

Loneliness is a subjective, distressing experience that results from perceived isolation or inadequate meaningful connections, where a discrepancy exists between a person’s preferred and actual relational experience (Prohaska *et al*. 2020). Research shows that loneliness is independently associated with poorer health outcomes, directly impacting morbidity and mortality; its effect on mortality is equivalent to smoking 15 cigarettes a day (Holt-Lunstad *et al*. 2010). Among young people, loneliness is linked with poor sleep, reduced life satisfaction, lower academic achievement, psychological stress, and poorer mental health, including anxiety, depression, and suicidal thoughts and behaviours (McClelland *et al*. 2020, Fardghassemi and Joffe 2022). These negative health effects make the causes of loneliness crucial to understand.

The widespread use of social media platforms among young people has made them an important digital health resource both for seeking and sharing health information (Zenone *et al*. 2021). Young people value social media’s simplicity, the perceived impartiality of health information, and access to relatable health stories from their peers (Lupton 2021, Montag *et al*. 2021). The abundance of data generated by these platforms makes them a data rich source to understand the experiences of loneliness among young people and an important area of health research.

Globally, TikTok is the fastest growing social media platform among young people (Lupton 2021). The hashtag #lonely had 13.7 billion views underscoring TikTok’s reach and ability to share health relevant information (TikTok 2023). It provides users with an algorithm generated stream of absorbing short-form videos; TikTok users engage with this content by watching, commenting, sharing, and liking, all of which contribute to the popularity and dominance of certain content. While TikTok offers an engaging experience for users, there is concern around the quality of health content on the platform, as well as the potential for exposure to sensitive content promoted by its algorithm, including portrayals of loneliness (Hill *et al*. 2023).

This study investigates the portrayal of loneliness on TikTok through a content analysis of the most popular videos under the #lonely hashtag at a point in time. We sought to: (i) understand how experiences of loneliness are portrayed on TikTok, (ii) assess the self-reported causes of loneliness on TikTok, and (iii) reflect on methodological opportunities and limitations for using TikTok to explore loneliness and similar health issues.

## METHODS

### Research design

Given the limited research on loneliness and TikTok, our objective was to perform an inductive content analysis which is well suited for exploratory qualitative investigations as it allows for open-ended and generative analysis. We followed a well-established, best practice method used to investigate TikTok content in many other studies (Herrick *et al*. 2021, Basch *et al*. 2022, Fowler *et al*. 2022, Chasca *et al*. 2023).

### Data collection

On 5 June 2023, we retrieved the URLs and corresponding metadata of TikTok videos under the #lonely hashtag using a TikTok scraper on the Apify data scraping platform (*n* = 904) (Apify 2023). The scraper collects publicly available videos and their associative metadata (views, likes, etc.) which appear on the TikTok desktop search function. While this does not retrieve all data under the #lonely hashtag, since TikTok limits access to the full range of its videos, it is currently aligned with best practices for sampling TikTok data in the absence of public data sharing or tools from TikTok itself. The sampled videos, furthermore, are typically the top-performing content under the hashtag at a point in time, therefore still representing dominant trends, and emulating the content a user would see if they searched the hashtag. All videos could be viewed by the general public.

We familiarized ourselves with the data, with one researcher watching all 904 videos and undertaking some initial open coding. A second researcher watched a 10% sample of these videos. This first viewing formed a preliminary understanding of the video content and relevance to the aims and objectives of the study. This informed the sampling strategy and proposed inclusion and exclusion criteria. We discussed initial findings and the inclusion and exclusion criteria were finalized.

### Inclusion and exclusion criteria

The inclusion criteria for the TikTok videos were

- In the English language.
- Publicly available and accessible on TikTok.
- With a personal human narrative (either spoken or via overlaid text) on the causes of loneliness.
- With a clear theme or portrayal of loneliness as the core subject.

The exclusion criteria for the TikTok videos were

- All non-English speaking videos.
- Television, film, music video, and videogame clips with no user narrative.
- People solely lip-syncing to songs.
- From a non-human perspective, e.g. animals.

### Final sample

The final sample contained 184 videos after removing duplicates and videos not meeting the inclusion criteria. To ensure inclusion reliability, a second reviewer watched a sample of these videos (*n* = 40). Any ambiguities and discrepancies were discussed and there was complete agreement between researchers about the final videos included. Based on previous studies of a similar nature, the number of videos was deemed an appropriate sample size for analysis (Herrick *et al*. 2021, Fowler *et al*. 2022). The collection and use of the data were also in accordance with TikTok’s terms and conditions. All included videos (*n* = 184) were manually downloaded, via scri.io, and assigned a unique number that corresponded with their metadata on Microsoft Excel.

### Data analysis

We developed a coding frame using an inductive approach. One researcher viewed all videos taking analytic notes to identify any patterns, narratives, unusual and striking content, and commonly used language. The code frame was applied to a selection of videos (*n* = 50), before refinements were made to the frame based on these observations; this process was repeated on another selection of videos (*n* = 50) before the coding frame was finalized. The videos were watched in the order they were downloaded by Apify to minimize potential bias introduced through manual selection.

During the coding process, which took place between June and August 2023, the two coders met routinely to discuss discrepancies, reach agreement on coding decisions, clarify ambiguous content, and discuss trends. Any videos with an element of ambiguity were marked and reviewed by the second researcher. Where two people or more were present in a video, it was coded from the perspective of the main person expressing a first-person perspective. To strengthen the reliability of the analysis, the second researcher watched and analysed a smaller sample of videos (*n* = 38). There was agreement on over 90% of coding decisions, showing high code application agreement.

In total, five characteristics were identified: (i) video tone, (ii) additional identities expressed in relation to loneliness, (iii) absences expressed as the cause of loneliness, (iv) unmet desires expressed as the cause of loneliness, and (v) changes in life circumstance as the cause of loneliness. A coding frame overview is seen in Table 1.

**Table 1.** Coding frame overview: research objective, codes, sub-codes.

Research objective	Code	Sub-codes
1	Video tone	(i) positive; (ii) negative; and (iii) neutral
1	Age Y/N	(i) <18 years; (ii) 18–30 years; (iii) 31–59 years; (iv) 60+ years; and (v) no age determined
1	Additional identities expressed in relation to loneliness Y/N	(i) mental health; (ii) depression; (iii) anxiety; (iv) disordered eating; (v) self-harm; (vi) suicidal ideation; (vii) family role (e.g. parent, child, sibling, and partner); (viii) LGBT; (ix) athlete; and (x) other
2	Absences expressed as the cause of loneliness Y/N	(i) no relationship (romantic, friendship, sibling, family); (ii) the absence of people at event/experience (birthday, Christmas, Friday night, night time, at home, other); (iii) quality of relationship (do not feel cared for, unreciprocated effort, do not feel enough, do not feel understood, feel let down, other); (iv) contact on social media; (v) physical touch; (vi) not living up to perceived expectation; and (vii) other
2	Unmet desires expressed as the cause of loneliness Y/N	(i) wanting a relationship (romantic, friendship, sibling, and family); (ii) to have people at a particular event/experience (birthday, Christmas, Friday night, nighttime, at home, other); (iii) online/social media contact; (iv) change in subjective experience of a relationship; (v) wanting physical touch; and (vi) other
2	Change in life circumstance as the cause of loneliness Y/N	(i) moving location; (ii) becoming a parent; (iii) romantic relationship breakdown; (iv) friendship breakdown; (v) death of significant other; (vi) COVID-19; (vii) growing up; and (viii) other

## RESULTS

### Key video characteristics

The videos in the sample (*n* = 184) received 687 625 500 views (average 3 490 485), 1 527 297 comments (average 7753), and 2 726 205 shares (average 13 839). It was not possible to determine age in 38.6% (*n* = 71) of videos. Where estimated age was determined (*n* = 113, 61.4%), most were under 17 years (*n* = 48, 26.1%), followed by 18–30 years (*n* = 35, 19%), then 31–59 years (*n* = 25, 13.6%), and lastly over 60 years (*n* = 5, 2.7%).

### Tone

An overall negative tone relating to loneliness was expressed by 83.2% (*n* = 153) of videos; 9.8% (*n* = 18) were positive; and 7.1% (*n* = 13) were neutral. Loneliness was suggested as a positive attribute by 1.1% (*n* = 2) of videos. These stated that lonely people were ‘strong enough to wait for what you deserve’ and had ‘created peace within their own company’. Combined, these two videos had over a million likes. A summary of the main findings is presented in Table 2.

**Table 2.** Results main findings and overview: characteristics, measure, and data examples.

Characteristics	Measure	Data examples
Overall video tone	*n* (%)	‘POV: me going home from school after faking my personality for 6 hours just to try and fit in (i can’t do this anymore)’
Negative	153 (83.2)	
Positive	18 (9.8)	
Neutral	13 (7.1)	
Additional identities expressed in relation to loneliness	*n* (%)	‘PPl [sic] my age having segs [sic] and doing drugs. Me: Hasn’t had my first kiss. #teen #crush #single #gay #bi #lgbt’
No	96 (52.2)	
Yes	88 (47.8)	
Additional identities expressed in relation to loneliness	*n* (%)	‘I’ve been starving myself carving my skin until my bones are showing’
Mental Health	48 (26)	
Depression	30 (16.3)	
Family role (e.g. parent, child, sibling, partner)	29 (16)	
Anxiety	11 (6)	
Depression and Anxiety	9 (4.9)	
Suicidal Ideation	9 (4.9)	
Other (e.g. only child, voter, carer, military spouse, disabled, autism, physical health)	6 (3.2)	
LGBT	4 (2.1)	
Athlete	3 (1.6)	
Disordered Eating	1 (0.5)	
Self-harm	1 (0.5)	
Absences expressed as the cause of loneliness	*n* (%)	‘My single ass seeing happy couples on tiktok just living their best lives and going on cute dates for the 10th time in a row’
Yes	128 (69.6)	
No	56 (30.4)	
Absences expressed as the cause of loneliness	*n* (%)	‘I stopped sending paragraphs, stopped begging, I stopped telling people how to treat me, and started walking away, blocking and distancing myself. Life may be lonely, but it’s becoming peaceful. Sometimes being alone is better than being surrounded by halfass [sic] people’.
No romantic relationship	28 (15.2)	
The absence of people at an event/experience	26 (14.4)	
No friendship	21 (11.4)	
No relationship generally	10 (5.4)	
Physical touch	6 (3.3)	
Contact on social media	3 (1.6)	
No family relationship	3 (1.6)	
No friendship and no relationship	3 (1.6)	
Poor relational quality as the cause of loneliness	*n* (%)	
General	22 (12)	
Friendship	17 (9.2)	‘When you spot a *20 person bachelorette party […] Did i go wrong somewhere in life?’
Relationship	15 (8.2)	
Family	7 (3.8)	
Online	1 (0.5)	
Quality of relationships	*n* (%)	‘it feels like you care about everyone but no one cares about you’
(Videos often cited more than once)		
Do not feel cared for	30 (16.3)	
Do not feel enough	20 (10.9)	
Unreciprocated effort	14 (7.6)	‘maybe I'm not meant for anyone and no ones meant for me and some ppl [sic] don't ever find their person’
Do not feel understood	13 (7.1)	
Other	13 (7.1)	
Feel let down	10 (5.4)	
Unmet desires expressed as the cause of loneliness	*n* (%)	‘out of 22 invites… no one came…’
No	157 (85.3)	
Yes	27 (14.7)	
Unmet desires expressed as the cause of loneliness	*n* (%)	‘So touch starved that I’m gonna hold hands with the next person who grabs a drink’
Wanting a relationship	13 (7.1)	
Wanting physical touch	13 (7.1)	
To have people at an event/experience	4 (2.1)	
More online contact	2 (1.1)	
Change in circumstance as the cause of loneliness	*n* (%)	‘You realized that everything you did for her didn’t make any difference’
No	143 (77.7)	
Yes	41 (22.3)	
Changes in circumstance as the cause of loneliness	*n* (%)	‘Little Miss thought it was finally going to be her turn but is once again wondering why she’s so easy to leave’
Romantic relationship breakdown	17 (9.2)	
Other	8 (4.3)	
Becoming a parent	3 (1.6)	
Death of significant other (including pet)	3 (1.6)	‘Since I moved to Oxfordshire i [sic] barely have any friends it can be lonely at times’
Friendship breakdown	3 (1.6)	
Moving location	3 (1.6)	
COVID-19	2 (1.1)	
Growing up	2 (1.1)	

### Expression of identity

**Figure 1.**
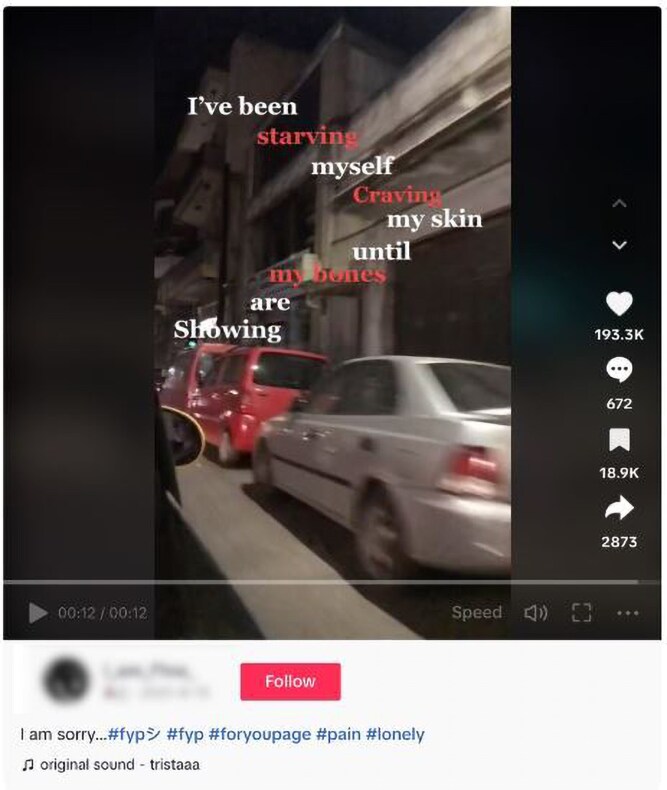
Image from TikTok video illustrating mental health and loneliness.

Around half of videos (*n* = 88, 47.8%) expressed an additional identity related to their experience of loneliness; either as an explanatory or associated factor. Mental health was the most discussed expression of identity (*n* = 48, 26%). Figure 1 provides an illustrative image of mental health and loneliness. Depression was the dominant subcategory of mental health (*n* = 30, 16.3%). Some mental health categories overlapped: 6% (*n* = 11) of videos referenced depression and anxiety, for instance.

Videos occasionally included sensitive content. Suicidal ideation was present in 4.9% (*n* = 9) of videos. Other mental health subcategories included self-harm (*n* = 1, 0.5%), disordered eating (*n* = 1, 0.5%), and bipolar personality disorder (*n* = 1, 0.5%).

Physical health was referenced in 0.5% (*n* = 1) of videos. 16% (*n* = 29) of videos identified the user’s family role as a cause of loneliness. These roles included parent (*n* = 11, 6%), child (*n* = 8, 4.3%), partner (*n* = 6, 3.2%), sibling (*n* = 4, 2.2%). Other expressions of identity associated with loneliness included Lesbian, Gay, Bisexual, Transgender (LGBT) (*n* = 4, 2.1%), and athlete (*n* = 3, 1.6%). Other categories referenced included a carer (*n* = 1, 0.5%), a voter (*n* = 1, 0.5%), being disabled (*n* = 1, 0.5%), being autistic (*n* = 1, 0.5%), and being a military spouse (*n* = 1, 0.5%). No person in any videos expressed an affiliation with a healthcare provider or stated that they were healthcare professionals.

### The self-reported causes of loneliness on TikTok

The self-reported causes of loneliness were broadly categorized into absence (*n* = 128, 69.6%), unmet desires (*n* = 27, 14.7%), and changes in life circumstances (*n* = 41, 22.3%).

### Absence

The most prevalent theme referenced as a cause of loneliness was absence (*n* = 128, 69.6%). The quality of relationships (*n* = 62, 33.7%) and a lack of relationships (*n* = 59, 32.1%) were most frequently expressed as the underlying causes of the absence, followed by an absence of people at an event or experience (*n* = 26, 14.1%), with birthdays most referenced (*n* = 6, 3.2%). For lack of relationship, the most keenly felt absence was romantic relationship (*n* = 28, 15.2%), followed by friendship (*n* = 21, 11.4%), then no relationship generally (*n* = 10, 5.4%).

**Figure 2.**
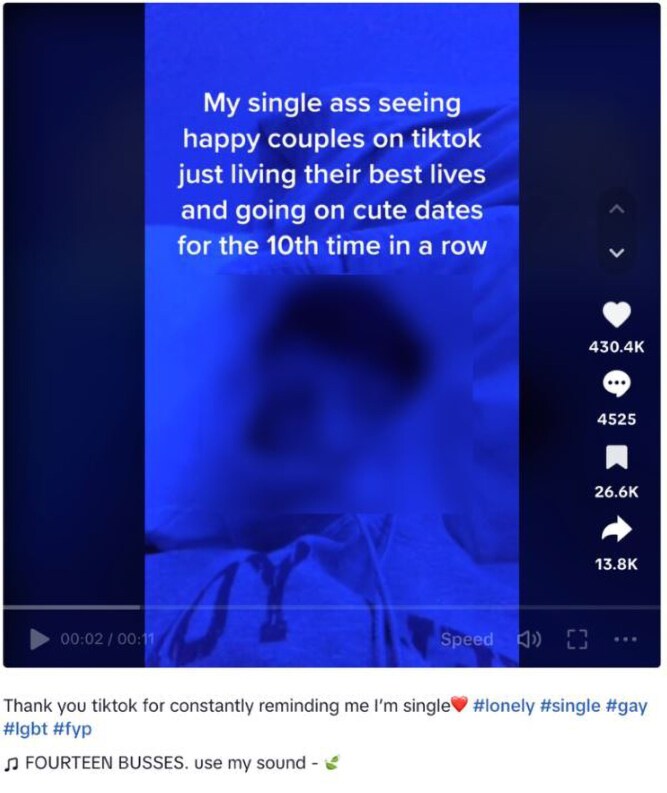
Image from TikTok video illustrating how the platform is used for comparing with peers.

Some videos expressed despair at the possibility of not finding a partner. For example, one young person said ‘maybe I'm not meant for anyone and no ones [sic] meant for me and some ppl [sic] don’t ever find their person’. While others expressed concern that not having relationships meant they did not matter to others and worried about the implications of this: ‘how long would it take for people to notice if you disappeared’. Seeing couples on social media was seen by some to draw attention to the absence in their own life, see Fig. 2.

There were emotive videos in the absence of quality relationships (*n* = 62, 33.7%) category, with individuals expressing deep frustration about this experience. The quality of relationships in general was referenced most (*n* = 22, 12%) followed by friendships (*n* = 17, 9.2%), romantic relationships (*n* = 15, 8.2%), and online relationships (*n* = 1, 0.5%).

Users expressed the absence of quality in their relationships in several ways, with many videos (*n* = 28, 15.2%) citing two or more reasons. The categories most referenced were: do not feel cared for (*n* = 30, 16.3%) and do not feel enough (*n* = 20, 10.9%). Examples from the data include: ‘it feels like you care about everyone but no one cares about you’; ‘[I have] never been anyone's first choice’; ‘[I am the] backup friend’.

These were followed by unreciprocated effort (*n* = 14, 7.6%) and not feeling understood (*n* = 13, 7.1%). One TikTok user shared: ‘One day your gonna [sic] meet someone who makes you realize there was never anything wrong with you’, demonstrating that the quality of relationships can affect how individuals feel about themselves. Another user was shouting in a video, clearly expressing frustration at people they encountered in life: ‘because I am SICK And […] tired Of wasting my GODDAMN TIME On people THAT NEVER GIVE TIME TO ME’. They went on to express how their dismay at past experiences impacted their approach to future relationships; demonstrating how loneliness could become entrenched.

**Figure 3.**
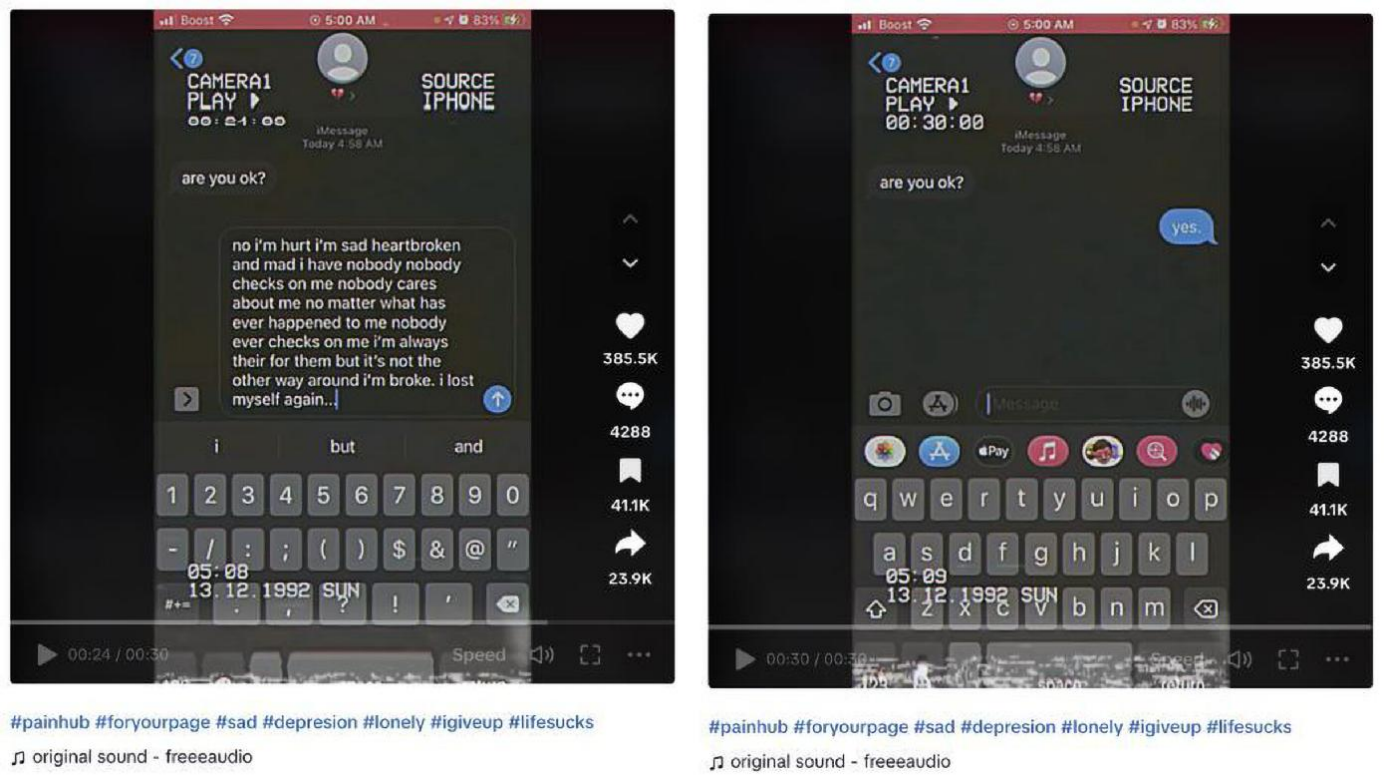
Images from TikTok video. Image on the left shows actual feelings; image on the right shows what they feel able to share.

Among other reasons given for the absence of quality in relationships was having to ‘mask’. This referred to a discord between how someone felt internally and what they felt able to share with others; see Fig. 3 showing two images taken in sequence from the same video. These images exemplify the discord expressed by users between how they actually feel and what they share with another person, though interestingly, they still feel able to share this on TikTok. Absence of contact on social media (*n* = 3, 1.6%) and absence of physical touch (*n* = 6, 3.3%) were other reasons given for loneliness.

Not living up to a perceived expectation emerged as a theme in the absence category. This was through comparing with others, be they friends, the opposite gender, or strangers, both in real life or online; or comparing to a social or cultural standard felt to be expected from a person in their position. Examples included beauty standards, standards of femininity, and expectations of sex and intimacy. One video captures the sentiment with the caption: ‘Where did I go wrong in life?’ and proceeds to compare their experience with others.

### Unmet desire

An unmet desire was expressed by 14.7% (*n* = 27) of videos as a cause of loneliness; with some (*n* = 10, 5.4%) also attributing it to an absence. The desires most sought after were wanting a relationship (*n* = 13, 7.1%) and wanting physical touch (*n* = 13, 7.1%). Of the videos desiring a relationship most wanted either a romantic relationship (*n* = 6, 3.3%) or a friendship (*n* = 6, 3.3%). One video attributed the difficulty of finding a partner to an over reliance on online matchmaking: ‘The number of young SINGLE men in America is at a record high […] it’s because of dating apps, that’s what’s ruining dating’. While another, with the #nofriendsclub hashtag, resigned to fulfil their desire for friends by ‘hang[ing] out with mum and dad’.

The videos desiring physical touch were a poignant reminder of the comfort of physical closeness offered by relationships. One video showed a person being hugged by a soft toy, while another showed a person in a shop with overlaid text stating: ‘So touch starved that I’m gonna hold hands with the next person who grabs a drink’.

### Change in circumstance

Change in life circumstance was expressed by 22.3% (*n* = 41) of videos as a cause of loneliness. Romantic relationship breakdown was referenced most (*n* = 17, 9.3%), followed by friendship breakdown (*n* = 3, 1.6%) becoming a parent (*n* = 3, 1.6%), the death of a significant other (*n* = 3, 1.6%), moving location (*n* = 3, 1.6%), the COVID-19 pandemic (*n* = 2, 1.1%), and growing up (*n* = 2, 1.1%). Other circumstances included being separated from children through divorce (*n* = 1, 0.5%), becoming wealthy (*n* = 1, 0.5%), being diagnosed with a chronic illness (*n* = 1, 0.5%), political beliefs (*n* = 1, 0.5%), and parents working new jobs (*n* = 1, 0.5%).

## DISCUSSION

This study aimed to analyse the sentiment of the top-performing content under the #lonely hashtag on TikTok, it found that: most videos referenced inadequate social connection as a cause of loneliness, reflecting the established literature; video content was overwhelmingly negative in tone; and no healthcare professionals sharing health content was in the dataset.

Results showed the expression of loneliness in this dataset reflected the established literature, namely driven by perceived inadequate social connection, classed most commonly as an absence (Fardghassemi and Joffe 2022, Wigfield *et al*. 2022). This is due to a discrepancy between actual and desired social relationships (Perlman and Peplau 1982), characterized here as poor quality or no relationships, seen in 69.6% of videos. Change in a person’s circumstances is another important cause of loneliness. In our sample, just over a fifth of videos identified that a change in circumstance was the source of loneliness; these included romantic relationship breakdown, the death of a loved one, and moving location. In 26% of videos, users associated mental health with loneliness reinforcing the association established in the literature (Twenge *et al*. 2019, Abi-Jaoude *et al*. 2020, Achterbergh *et al*. 2020, Sha and Dong 2021). Depression was the most frequently mentioned mental health challenge, appearing in 16.3% of videos. Suicidal ideation was referenced in 4.9% of videos, a finding chiming with the literature recognizing that loneliness can be a predictor for later suicidal ideation and/or behaviour (McClelland *et al*. 2020). The social stigma related to being lonely also, no doubt, contributed to the need for anonymity among users, making it unsurprising that in over a third of videos (38.6%) age could not be determined. Allowing users to post anonymously is an appealing aspect of TikTok and enables a more complete expression of loneliness than found through traditional approaches. The results of this novel research method corroborate traditional literature showing its potential for future research on comparable health issues.

The emotive nature of loneliness is effectively captured in this study’s dataset. The candid videos offer a compelling insight into the most relatable portrayals of loneliness on TikTok. 83.2% of videos have a negative tone, demonstrating the popularity of such content. This is unsurprising given that video virality is reliant on factors like the emotional aspect of content, including its polarity and emotional charge (Tsugawa and Ohsaki 2017). As the experience of loneliness is heavily shaped by cultural norms, the top-performing videos also reflect the most culturally acceptable experiences of loneliness today (The Lancet 2023). The overwhelming negative tone suggests that this type of content is more likely to generate engagement, while TikTok’s algorithm further amplifies this trend, as users have no control over what is displayed to them. This drives the loneliness discourse on TikTok and perpetuates a cycle of negative content.

The absence of health professionals sharing positive health information for managing loneliness, given the overall negative discourse, is worrying but expected. It reflects a trend found in previous mental health research on TikTok where only 16% of videos shared coping strategies (Basch *et al*. 2022). This lack of health information under the hashtag #lonely is an important finding, particularly as social media platforms are widely recognized as an important source of health information for young people (Zenone *et al*. 2021). It shows either a lack of loneliness-related health information on the platform, or that existing health information is ineffective in enticing engagement and reaching large audiences. Existing research on the health impact of positive online health messaging for young people is limited; and is likely a contributing factor to a reticence among health organizations to promote online health information on loneliness. Identifying effective, engaging, and evidence-based health information that positively impacts the loneliness of TikTok users should therefore be a future research priority (Nesi 2020).

The platforming of sensitive content, as found among this dataset, acts as an important reminder that social media companies have a role in safeguarding their, mostly young, users (Lupton 2021). TikTok has been compelled to issue well-being guides to support optimal messaging on the platform specifically around mental health (Basch *et al*. 2022). Such support is particularly important on TikTok as users have no control over content they see, leaving them unprepared and vulnerable to the detrimental effects of sensitive content (Sha and Dong 2021). Those sharing such content may require support if their video becomes viral or they are subject to adverse comments. This is a pressing priority given online services are known to amplify harmful content (Regehr *et al*. 2024).

Whether sharing these personal accounts on TikTok, and the engagement that follows, alleviates loneliness is beyond the scope of this study. The appeal to share may lie in the social media companies’ effective exploitation of the human fear of being alone, with loneliness barely existing on the platforms as someone is always available and willing to comment and engage (Bauman and Lyon 2013). This may account for the limited reference to social media (1.6%) as a cause of loneliness in the dataset. Despite the volume of communication on social media there is, perhaps, little substantial and sustaining dialogue (Bauman and Lyon 2013). This threatens to perpetuate a user’s loneliness, especially as meaningful connections are considered its most effective antidote (Segrin and Passalacqua 2010).

Our study has some limitations such as the inclusion of English-only videos; the omission of video comments from the analysis; and the exclusion of other related hashtags (e.g. #loneliness) from the dataset. However, content tagged under #loneliness, for instance, is unlikely to be markedly different from that tagged under #lonely. Many videos on TikTok have no hashtag at all, some videos with content focused on loneliness will therefore be missed from the dataset. The absence of gender and ethnicity data is a limitation of this study, as it restricts our ability to explore how experiences of loneliness may differ across social identities. However, we omitted these variables to avoid making assumptions based on visual content, which could misrepresent individuals and reinforce bias. Focusing solely on English-language videos risks underrepresenting views of important groups with diverse perspectives and experiences, particularly impacting culturally and linguistically diverse communities and those from low- and middle-income countries. Other social media platforms may furthermore offer different insights into the experience of loneliness and its online portrayal, especially those that rely on existing social networks like Facebook. Similarly, those who use and post on TikTok may represent a self-selecting group who do not accurately reflect wider population trends.

## CONCLUSION

Loneliness is a serious public health issue that has worsened among young people in recent years. Considerable social media use in this group has made it an essential forum for discussing personal health experiences and finding health information. Most loneliness-related videos in this study portrayed the issue negatively, with few offering advice on ways to cope, and none originating from healthcare professionals or healthcare organizations.

Future research should focus on the effectiveness of health messaging related to loneliness on TikTok, including an examination of health content creators, content integrity, user reactions, and overall effectiveness. Additionally, researching how users incorporate information from TikTok into their daily lives and identifying the most impactful health sources is crucial. Ensuring accessible and transparent research tools on TikTok is vital to advancing this effort.

Most videos indicated that an absence of relationships, either due to their poor quality or non-existence, was the primary cause of loneliness. This is consistent with the existing research recognising loneliness as a gap between the desire and reality of social connectedness. The emotive nature of the videos in the dataset highlights the type of media that gains traction on the platform. The need for TikTok to exercise a duty of care and safeguard users, both sharing and being shown intimate and sensitive content, on their platform is clear. As is the need for healthcare organizations to provide support through evidence-based health advice for TikTok users experiencing loneliness.

## Data Availability

Available from the corresponding author on reasonable request.
